# PROTOCOL: The methodological and reporting characteristics of Campbell reviews: a methodological systematic review

**DOI:** 10.1002/cl2.1010

**Published:** 2019-06-24

**Authors:** Xiaoqin Wang, Vivian Welch, Liang Yao, Julia H. Littell, Huijuan Li, Meixuan Li, Nan Yang, Jianjian Wang, Larissa Shamseer, Yaolong Chen, Kehu Yang, Jeremy M. Grimshaw

**Affiliations:** ^1^ Evidence‐Based Medicine Centre, School of Basic Medical Sciences Lanzhou University Lanzhou Gansu China; ^2^ Centre of Implementation Research Ottawa Hospital Research Institute Ottawa Canada; ^3^ Bruyere Research Institute Ottawa Ontario Canada; ^4^ Clinical Division, School of Chinese Medicine Hong Kong Baptist University Kowloon Tong Hong Kong China; ^5^ Graduate School of Social Work and Social Research Bryn Mawr College Bryn Mawr Pennsylvania; ^6^ Centre for Journalology, Clinical Epidemiology Program Ottawa Hospital Research Institute Ottawa Ontario Canada; ^7^ School of Pharmacy University of Maryland Baltimore Maryland

## 1 BACKGROUND

Systematic reviews aim to “sum up the best available research on a specific question by synthesizing the results of several studies” (Campbell Collaboration, 2018). They use transparent procedures to find, evaluate, and synthesize the results of relevant research whilst minimizing bias. They are increasingly popular across a wide range of sectors to inform policy and practice. Systematic reviews can support policymakers to develop evidence‐informed policy and help practitioners to keep up‐to‐date with relevant content knowledge (IOM, 2011; Oliver, 2015). In addition, granting agencies increasingly require the use of systematic reviews to justify new research. The trustworthiness of a systematic review is dependent upon the extent to which the review authors conducted the review using robust methods and the quality of reporting of the methods of the review (Steffen, 2010).

The Campbell Collaboration undertakes systematic reviews of the effect of social and economic policies to help policymakers, practitioners, and the public to make well informed decisions about policy interventions (Welch, 2018). It has established a number of policies and procedures to promote rigorous methodology and transparent reporting of Campbell reviews, for example, the Methodological Expectations of Campbell Collaboration Intervention Reviews (MECCIR) were introduced in 2014 (MECCIR, 2018). However, we know little about how Campbell reviews are conducted and reported. In the health field, Moher, Tetzlaff, Tricco, Sampson, and Altman (2007) and Page et al. (2016) have demonstrated poor conduct and highly variable reporting of systematic reviews. For example, only 7% of the included systematic reviews searched sources of unpublished data, the risk of publication bias was considered in less than half of systematic reviews, and the reporting quality was highly variable (Page et al., 2016). In social science, the American Psychological Association released reporting standards for psychological qualitative research, which are also useful for a broad range of social sciences (Levitt et al., 2016). In 2017, a study examined the reporting of the method section of quantitative systematic reviews in the field of industrial and organizational psychology and found the reporting quality on methods is insufficient (Schalken & Rietbergen, 2017). For example, time period covered by the search was only fully reported in 23.3% (28) reviews. To date, however, there has not been a comprehensive review of the methods and reporting of Campbell reviews.

## 2 OBJECTIVES

The review has three main objectives

(1) To assess the methodological quality of Campbell reviews.
(2) To assess the reporting characteristics of Campbell reviews.
(3) To compare the methodological and reporting characteristics of Campbell reviews published from 2011 January to 2014 September and 2014 October (when the MECCIR was adopted) to 2018 January.

The review will also compare the methodology and reporting characteristics of

(1) Campbell reviews with versus without coregistration in Cochrane Library.

## 3 METHODOLOGY

### 3.1 Eligibility criteria

Completed Campbell systematic reviews of the effects of interventions published between January 2011 to January 2018. We will only include the interventional reviews, while others like reviews about predictors will be excluded. We will exclude records where only the protocol not the final systematic review is published. We will include the most recent version of updated reviews.

To ensure the comparability, we assessed the number of eligible reviews to assess the feasibility of subgroup analyses, where there will be 97 eligible reviews in total: 45 published from 2011 January to 2014 September and 53 published from 2014 October to 2018 January; reviews are from each five coordinating groups including crime and justice (21), education (26), international development (28) and welfare (37), nutrition (1); 74 were registered on Campbell only and 23 were coregistered with Cochrane.

### 3.2 Search strategy

We will search the Campbell Library to identify all the completed intervention reviews published from January 2011 to January 2018.

### 3.3 Data extraction

Our abstraction form was developed based on the Preferred Reporting Items for Systematic Reviews and Meta‐Analyses (PRISMA) guidelines (Moher, Liberati, Tetzlaff, Altman, & PRISMA Group, 2009); the A MeaSurement Tool to Assess systematic Reviews 2 (AMSTAR‐2) instrument (Shea et al., 2017); mandatory reporting items for methods and results from the MECCIR reporting standards Version 1.1 (MECCIR, 2018); and additional review characteristics identified in similar methodological studies of reporting quality (Page et al., 2016).

The draft data abstraction form included 85 items. Following discussion within the review team we excluded 10 items to reduce repetition within the data abstraction form and to focus on higher level items resulting in the final data abstraction form of 75 items.

The following data will be collected

(1) Basic information, including publication year, number and institute of authors, update status, coregistration information, coordinating group, focus of the review, types of intervention, source of funding, and declaration of interest of authors.
(2) Methodological characteristics reported in the review, including protocol preparation, data sources and search strategies, selection of studies, data collection, data analysis, number of outcomes specified in the results, and assessment of the risk of bias.
(3) Results characteristics reported in the review, especially the results corresponding to the methods, including number of records retrieved and included, result of the analysis and assessment.
(4) Discussion characteristics reported in the review and conclusion, including subheadings used, limitations at the study level and review level, implication for practice and future research, and so on.

Data extraction will consider all documents relevant to the completed review including the protocol and full review. Data will be abstracted using a standardized extraction form in Microsoft Excel 2018.

All extractors will independently pilot‐test the form. Reviewers will abstract two reviews and a third reviewer (X.W.) will check all the data and consult with a fourth reviewer (J. M. G.) when necessary. We will conduct further rounds of pilot testing until acceptable levels of agreement are reached. Subsequently, data from each review will be independently extracted by two independent reviewers, and any discrepancies in the data extracted will be resolved via discussion or adjudication by a third reviewer (X. W.) if necessary.

### 3.4 Data synthesis

We will use descriptive statistics (frequencies and percentages) to describe reporting characteristics of systematic reviews.

### 3.5 AMSTAR‐2

Individual AMSTAR 2 items will be categorized as yes, partial yes, no; for items concerning meta‐analysis, we have added a “no meta‐analysis conducted” response (Appendix 2). The overall rating of confidence in the results of each review will be clarified as high, moderate, low, critically low (Box 1) according to the seven critical domains: (1) protocol registered before commencement of the review (item 2); (2) adequacy of the literature search (item 4); (3) justification for excluding individual studies (item 7); (4) risk of bias from individual studies being included in the review (item 9); (5) appropriateness of meta‐analytical methods (item 11); (6) consideration of risk of bias when interpreting the results of the review (item 13); and (7) assessment of presence and likely impact of publication bias (item 15).

Box 1  

**High**

No or one noncritical weakness: The systematic review provides an accurate and comprehensive summary of the results of the available studies that address the question of interest.

**Moderate**

More than one noncritical weakness*: The systematic review has more than one weakness but no critical flaws. It may provide an accurate summary of the results of the available studies that were included in the review.

**Low**

One critical flaw with or without noncritical weaknesses: The review has a critical flaw and may not provide an accurate and comprehensive summary of the available studies that address the question of interest.

**Critically low**

More than one critical flaw with or without noncritical weaknesses: The review has more than one critical flaw and should not be relied on to provide an accurate and comprehensive summary of the available studies.

As the first comprehensive analysis of the reporting and methodological quality of Campbell review, this study will help the Campbell Collaboration, Campbell Coordinating Groups, and authors to identify areas for improvement. It can also benefit the potential users of Campbell reviews by providing an up to date assessment of the quality of Campbell reviews.

For both the methodology (AMSTAR‐2 assessment) and the reporting of Campbell reviews (PRISMA and MECCIR reporting standards), we will use Stata version 12 to compare the quality of reviews: (a) published before MECCIR (before 2014 September) and after MECCIR (after 2014 October) and (b) that were Campbell registered only versus coregistered on Campbell and Cochrane. Associations will be quantified using the risk ratio, with 95% confidence intervals.

## ACKNOWLEDGMENTS

Xiaoqin Wang is supported by the China Scholarship Council. Vivian Welch is supported by an Ontario Early Researcher Award (2014–2019). Julia Littell is supported by by Bryn Mawr College. Jeremy Grimshaw is supported by a Canada Research Chair in Health Knowledge Transfer and Uptake.

Kehu Yang is supported by Evidence Based Social Science Research Center of Lanzhou University (Project ID: LZDX‐EBSS‐2018001).

## CONFLICT OF INTERESTS

Vivian Welch is the editor in chief of the Campbell Collaboration, Jeremy Grimshaw is the Chair of the board of the Campbell Collaboration, and Julia Littell is a member of the Technical Advisory Group of the Campbell Collaboration.

## AUTHOR CONTRIBUTIONS

Content: X. W., J. G. Systematic review methods: V. W., J. G., J. L., K. Y., L. S. Systematic review methods: V. W., J. G., J. L., K. Y., L. S. Statistical analysis: X. W., L. Y., Y. C. Information retrieval: X. W., L. Y., J. W., X. L., H. L., M. L.
